# Recent Advances in Statistical Data and Signal Analysis: Application to Real World Diagnostics from Medical and Biological Signals

**DOI:** 10.1155/2016/1643687

**Published:** 2016-08-02

**Authors:** Dwarikanath Mahapatra, Krishna Agarwal, Reza Khosrowabadi, Dilip K. Prasad

**Affiliations:** ^1^IBM Research, Melbourne, VIC, Australia; ^2^Singapore-MIT Alliance for Research and Technology, Singapore; ^3^Shahid Beheshti University, Tehran, Iran; ^4^Nanyang Technological University, Singapore

Medical and biological signals span almost the entire spectrum from EEG to X-rays and their sources range from molecular scales to large organs such as heart, brain, and muscles. Signal processing techniques (including image analysis) are constantly serving towards improving the state-of-the-art in medical and biological data analysis and interpretation. There is constant scientific endeavour to get better insight into the hidden information beneath the huge stack of medical data that we encounter. Consequently there has been a major shift towards quantitative analysis of medical data through various computational approaches. Computational approaches that have been hugely popular and found important applications include computational modeling, Bayesian and graphical models, machine learning, deep-learning, pattern recognition, optimization, spectral and pseudospectral analysis, stochastic modelling, iterative system model adaptation, and multiscale multiphysics analysis to name a few.

This special issue was an attempt to bring together interesting works that use advanced statistical techniques for cutting edge medical applications and biological signals for disease detection and diagnosis. We received submissions from a wide range of approaches and applications such as biological/medical image and signal processing, sensor and probe's signal analysis, imaging and microscopy techniques, human brain mapping, modeling and simulation of biological, biochemical, cellular, and subcellular processes, sensor fusion, wearable devices based health informatics, histopathology image analysis, and brain computer interface in medicine.

We have selected papers which were particularly relevant to bringing forward methods and applications that we think will be interesting for a wide range of researchers. Out of 14 submissions we selected 6 manuscripts. We hope that this selective publication process would prove beneficial for those researchers who wish to advance the state-of-the-art in medical imaging research.

*Dwarikanath Mahapatra* *Dwarikanath Mahapatra* *Krishna Agarwal* *Krishna Agarwal* *Reza Khosrowabadi* *Reza Khosrowabadi* *Dilip K. Prasad* *Dilip K. Prasad*

